# RBC Barcoding Allows for the Study of Erythrocyte Population Dynamics and *P. falciparum* Merozoite Invasion

**DOI:** 10.1371/journal.pone.0101041

**Published:** 2014-07-01

**Authors:** Martha A. Clark, Morgan M. Goheen, Nicholas A. Spidale, Raj S. Kasthuri, Anthony Fulford, Carla Cerami

**Affiliations:** 1 Department of Microbiology & Immunology, University of North Carolina at Chapel Hill, Chapel Hill, North Carolina, United States of America; 2 Division of Hematology, University of North Carolina at Chapel Hill, Chapel Hill, North Carolina, United States of America; 3 Department of Epidemiology, University of North Carolina at Chapel Hill, Chapel Hill, North Carolina, United States of America; 4 Medical Research Council International Nutrition Group, London School of Hygiene and Tropical Medicine, London, United Kingdom & Medical Research Council Keneba, The Gambia; Bernhard Nocht Institute for Tropical Medicine, Germany

## Abstract

*Plasmodium falciparum* invasion of host erythrocytes is essential for the propagation of the blood stage of malaria infection. Additionally, the brief extracellular merozoite stage of *P. falciparum* represents one of the rare windows during which the parasite is directly exposed to the host immune response. Therefore, efficient invasion of the host erythrocyte is necessary not only for productive host erythrocyte infection, but also for evasion of the immune response. Host traits, such as hemoglobinopathies and differential expression of erythrocyte invasion ligands, can protect individuals from malaria by impeding parasite erythrocyte invasion. Here we combine RBC barcoding with flow cytometry to study *P. falciparum* invasion. This novel high-throughput method allows for the (i) direct comparison of *P. falciparum* invasion into different erythrocyte populations and (ii) assessment of the impact of changing erythrocyte population dynamics on *P. falciparum* invasion.

## Introduction

Malaria is responsible for significant morbidity and mortality in the developing world, causing an estimated 250 million infections and 1 million deaths annually[1]. Malaria can be caused by any of several different plasmodia species capable of infecting humans; of these, *Plasmodium falciparum* is the most malignant. Upon transmission to the human host by an Anopheles mosquito, plasmodial sporozoites migrate to and infect liver hepatocytes. Following the asymptomatic liver stage, merozoites are released into the bloodstream where they infect host red blood cells (RBCs). It is the RBC stage of infection that is responsible for all symptoms of disease. Upon entry into the host RBC the malaria parasite progresses through a 48 hour lifecycle. At the end of the 48 hour intra-erythrocytic lifecycle the host RBC is ruptured and newly formed merozoites are released into the serum to invade new host RBCs [2]. The extracellular merozoite stage of the malaria parasite is very brief. Invasion occurs within 10 minutes of RBC rupture and the 48 hour intra-erythrocyte cycle begins again [3]. Successful invasion of the host RBC is essential for propagation of the parasite. Additionally the merozoite is one of the only parasite stages directly exposed to the host immune system. As a result, merozoite invasion represents a promising point of attack for antimalarial drugs and vaccines [4].

The RBC population of a given individual is heterogeneous; RBC age, anemia, acidosis, infection, and Glucose-6-phosphate dehydrogenase (G6PD) deficiency are examples of a few conditions which can contribute to heterogeneity in an individual's RBC population [5]–[9]. Therefore, the most relevant approach for studying how RBC heterogeneity influences *P. falciparum* invasion would be to combine different RBC populations in a single culture condition and study subsequent *P. falciparum* infection. In addition, direct comparison of *P. falciparum* invasion into different “target” RBC populations would allow for increased sensitivity to small differences that may exist between parasite invasion of different RBCs. The diversity and redundancy that exists in both host RBC ligands and merozoite invasion receptors makes accuracy in the measurement of invasion important [4], [10], [11].

Flow cytometry has revolutionized the study of malaria; increasing the throughput, sensitivity, and accuracy of many malaria assays [12]. Pattanapanyasat et al. was the first to use the power of flow cytometry to distinguish two discrete RBC populations in a single culture condition [13], [14]. Since only one of the two RBC populations was labeled, the approach taken by Pattanapanyasat et al. does not account for “contaminant” RBCs present in the inoculum. Moreover, Pattanapanyasat et al. examined overall growth of *P. falciparum* and not invasion. Previous groups have dealt with “contaminant” RBCs by enzymatically treating the parasitized RBC (pRBC) inoculum to remove parasite invasion ligands from uninfected RBCs present in the inoculum [15], [16]. Alternatively, a two-color flow cytometry based invasion assay developed by Theron et al. addresses the issue of “contaminant” RBCs by labeling “target” RBCs with CellTrace dyes, making “contaminant” and “target” RBCs clearly distinguishable from one another [17]. Neither the enzyme treatment of inoculum RBCs nor the two-color approach allow for the direct comparison of *P. falciparum* invasion into different “target” RBC populations contained in the same well.

Here we utilize fluorescent RBC barcoding to build upon the methods described by Theron et al.[17] and Pattanapanyasat et al.[14]. RBC barcoding allows direct comparison of *P. falciparum* invasion into two distinct “target” RBC populations in the same well. This high-throughput approach provides increased sensitivity for detecting differences in merozoite invasion as well as a method for studying how different RBC populations interact to determine malaria infection.

## Materials and Methods

### *P. falciparum* culture

*P. falciparum* parasite lines 3D7 (MR4, MRA-102), Dd2 (MR4, MRA-156), and FCR3-FMG/Gambia (MR4, MRA-736) were routinely cultured in O positive (O+) RBCs within two weeks of being obtained from healthy individuals. RBCs were collected at the Clinical and Translational Research Center at the University of North Carolina, Chapel Hill and their use for this study was approved by the Institutional Review Board at the University of North Carolina at Chapel Hill (IRB# 09-0559). Written consent was obtained from all donors using a consent form specifically approved by the IRB. Cultures were maintained at 2% hematocrit in complete media containing RPMI 1640 with 10% AlbuMAX II, 1 mM hypoxanthine, 20 mM L-glutamine, 0.45% glucose, and 10 µg/L gentamicin (ACM). Cultures were shaken at 37°C in 5% O_2_, 5% CO_2_ and 90% N. Parasite density was maintained between 0.5% and 10% *P. falciparum* parasitized RBCs (pRBCs). pRBC cultures were synchronized using 5% D-sorbitol to select for ring stage parasites, followed by Magnetic Activated Cell Sorting (MACS) (Miltenyi Biotec) isolation of hemozoin containing trophozoite and schizont stage pRBCs 24 hours later [18].

### Barcoded RBC invasion assay

Briefly, O+ RBCs were labeled at 2% hematocrit in RPMI with 5 µM of either CellTrace Violet (RBC^Violet^) or DDAO (RBC^DDAO^) (Invitrogen) for two hours with shaking at 37°C, washed twice with ACM, incubated 30 minutes in ACM with shaking at 37°C, and finally washed twice with RPMI and stored in RPMI at 4°C. DDAO and Violet fluorescent intensity varied between experiments. To confirm distinct separation of the fluorescent signals of RBC^unlabeled^, RBC^DDAO^, and RBC^Violet^, each was analyzed by flow cytometry alone and in combination. Invasion assays were performed with fluorescently labeled RBCs within two days of labeling. For barcoded RBC invasion assays, RBC^DDAO^ and RBC^Violet^ were counted using a Nexcelom Bioscience Cellometer Auto 2000 and 2×10^7^ total RBCs were delivered in triplicate into a 96 well plate. MACS purified pRBCs from routine cultures were counted using a Nexcelom Bioscience Cellometer Auto 2000 and 2×10^5^ pRBCs were added to each well. Experiments were maintained for 18–24 hours under standard culture conditions to allow for schizont rupture and subsequent invasion of RBC^DDAO^ and RBC^Violet^. Following merozoite invasion, cells were stained with 1× DNA dye SYBR Green I (Invitrogen), fixed with 1% paraformaldehyde and 0.0075% glutaraldehyde in Alsever's Solution (Sigma) as described previously[19], and analyzed by flow cytometry or microscopy. A small (0.16%) double positive Violet and DDAO RBC population – expected to be RBC doublets that escaped singlet gating – was occasionally observed and was excluded from the analysis. We have confirmed that dye is not transferred between RBCs during the course of barcoded RBC invasion experiments (data not shown).

### Enzyme treatment of RBCs

RBCs were treated with trypsin, chymotrypsin, or neuraminidase in accordance with the Sanger Institute flow cytometry-based invasion phenotyping protocol (http://www.sanger.ac.uk/research/projects/malariaprogramme-rayner/sop-flow-cytometry-invasion-assay.pdf). Briefly, RBCs labeled with either CellTrace Violet or DDAO were treated with 0.02 U/mL neuraminidase (Sigma), 50 µg/mL trypsin (Sigma), or 0.91 mg/mL chymotrypsin (Sigma) at 2% hematocrit in RPMI for 1 hour with shaking at 37°C, washed twice with ACM, incubated 30 minutes in ACM with shaking at 37°C, and finally washed twice with RPMI. Enzyme treated RBCs were stored in RPMI at 4°C for up to 1 day before being used in experiments. Untreated RBC^DDAO^ and RBC^Violet^ were combined and included as a control for all experiments.

### Microscopy

pRBC^DDAO^ and pRBC^Violet^ were stained with 1× DNA dye SYBR Green I, fixed with 1% paraformaldehyde and 0.0075% glutaraldehyde, and then viewed by oil immersion with a 63X/1.4 numerical aperture Oil Plan Apo objective lens on a Zeiss CLSM 710 Spectral Confocal Laser Scanning Microscope. Images were captured with Zeiss ZEN 2011 software.

### Flow cytometry analysis

Flow cytometry was performed at the UNC Flow Cytometry Core Facility on a Beckman-Coulter (Dako) CyAn ADP cytometer. Channels and probes used on the CyAn ADP Cytometer included: CellTrace Violet (405 nM excitation, 450/50 bandpass), SYBR Green I (488 nm excitation, 530/40 bandpass), and CellTrace DDAO (635 nm excitation, 665/20 bandpass). Detector gain settings varied between experiments to optimize signal but were kept constant within individual experiments and spectral overlap compensation was not necessary with this configuration. *P. falciparum* pRBCs were gated on SYBR Green I signal. Dako Cyan data was collected and analyzed with Summit v4.3.01. Linear amplification of forward scatter was used to set event threshold in order to exclude cell debris, microparticles and doublets. For all experiments samples were diluted to 0.001–0.002% haematocrit and 100,000–500,000 total events were collected.

### Data analysis and statistical methods

All experiments were performed in triplicate. Results are from either one representative experiment of at least three independent experiments or the combined results of at least three independent experiments. To compare the relative susceptibility of two different RBC populations to *P. falciparum* invasion, we determined the susceptibility index (SI) with an unadjusted Odds Ratio (performed with Stata/IC, v10, Stata Corp, 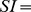 College Station, TX) as shown in the equation: 
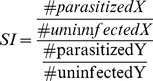



The SI represents the relative risk of the two RBC populations (represented by X and Y in the equation) to *P. falciparum* invasion. For experiments comparing*P. falciparum* invasion of RBC^DDAO^ and RBC^Violet^, X = RBC^DDAO^ and Y = RBC^Violet^. For experiments comparing *P. falciparum* invasion of untreated (RBC^Ø^) and enzyme treated (RBC^N^, RBC^T^, or RBC^C^), X = enzyme treated RBC and Y =  RBC^Ø^. An SI of 1.0 indicates no difference in *P. falciparum* invasion of the two RBC populations. *P. falciparum* invasion rate is the number of RBC invasions per inoculated pRBC. Linear regression was employed to investigate the associations between RBC enzyme treatment and parasite invasion *in vitro* using the number of each RBC population as the independent variable and total invasions/1×10^5^ RBCs as the dependent variable. Analysis of covariance (ANCOVA) was conducted to determine whether the invasion into the two groups were the same. An alpha of 0.05 was set a priori to determine statistically significant differences.

## Results

### Barcoding RBCs with CellTrace dyes DDAO and Violet allows for the direct comparison of *P. falciparum* invasion into different RBC populations

**Figure 1:**
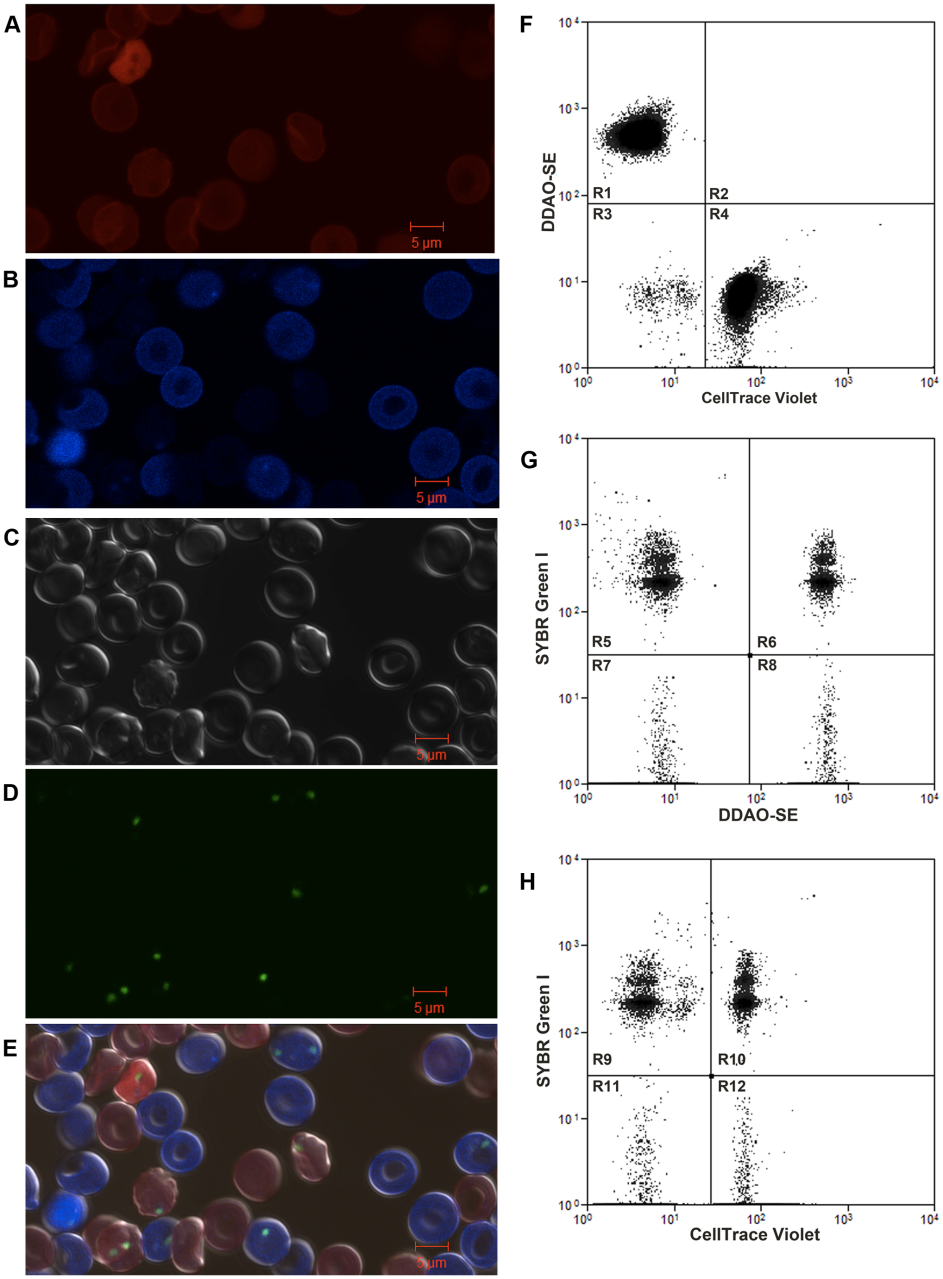
RBCs barcoded with CellTrace DDAO and Violet can be combined to directly compare *P. falciparum* invasion in an invasion assay. RBCs were labeled with 5 µM of either DDAO (A) or CellTrace Violet (B). Cells were then combined and infected with MACS purified unlabeled pRBCs. Experiments were incubated 18–24 hours. Cells were then stained with DNA dye SYBR Green I, fixed, and examined by brightfield (C) and fluorescence microscopy (D, E) and by flow cytometry (F–H). (A) shows red channel only, (B) shows violet channel only, (C) shows brightfield, (D) shows green channel only and (E) shows merge of red, violet, and green channels. (F) Flow cytometry plot of RBCs stained with CellTrace DDAO (R1) and CellTrace Violet (R4) and non-stained pRBC (R3). (G) Flow cytometry plot shows DDAO negative pRBCs (R5), DDAO negative uninfected RBCs (R7), DDAO positive pRBCs (R6) and DDAO positive uninfected RBCs (R8). (H) Flow cytometry plot shows CellTrace Violet negative pRBCs (R9), CellTrace Violet negative uninfected RBCs (R11), CellTrace Violet positive pRBCs (R10) and CellTrace Violet positive uninfected RBCs (R12).

**Figure 2:**
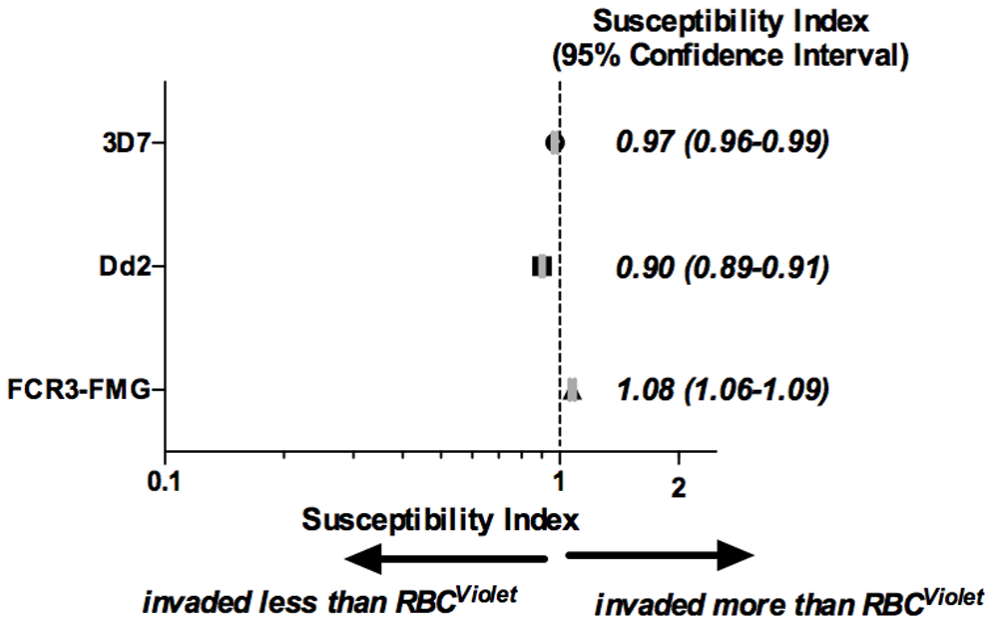
Direct comparison of *P. falciparum* strains 3D7, Dd2 and FCR3-FMG invasion into RBCs barcoded with DDAO and CellTrace Violet. Equal numbers (1×10^7^) of RBCs stained with either 5 µM CellTrace DDAO (RBC^DDAO^) or CellTrace Violet (RBC^Violet^) were combined (total of 2×10^7^ RBCs per well) and inoculated with 2×10^5^ MACS purified trophozoite stage *P. falciparum* strains 3D7, Dd2 or FCR3-FMG. Invasion experiments were incubated 18–24 hours to allow for rupture of schizonts and subsequent invasion of merozoites into labeled RBCs, then stained with DNA dye SYBR Green I (to identify pRBCs), fixed and analyzed by flow cytometry. The Susceptibility Index (SI), an unadjusted Odds Ratio assessing the relative risk of RBC^DDAO^ and RBC^Violet^ to 3D7, Dd2, and FCR3-FMG invasion. The marker represents the SI point estimate and the bar represents the 95% confidence interval (CI). A SI of 1.0 indicates no difference in parasite invasion of two RBC populations. Data is the combination of four, seven, and three independent experiments performed in triplicate with 3D7, Dd2, and FCR3-FMG respectively.

To develop a *P. falciparum* RBC invasion assay that would allow for direct comparison of invasion of *P. falciparum* into two different RBC populations, we utilized a fluorescent RBC staining approach that permits the definitive detection of two distinct RBC populations by flow cytometry. DDAO was used to label the first RBC population (RBC^DDAO^) (**Figure 1A**) and CellTrace Violet was used to label the second RBC population (RBC^Violet^) (**Figure 1B**). As has been reported with CellTrace DDAO [17], CellTrace Violet does not affect *P. falciparum* RBC invasion (**Figure S1**). We next examined whether RBC^DDAO^ could be combined with RBC^Violet^ in the barcoded RBC invasion assay. An equal number of RBC^DDAO^ and RBC^Violet^ were combined, inoculated with Magnetic Activated Cell Sorting (MACS) purified trophozoite stage pRBCs and incubated for 18–24 hours to allow for schizont rupture and subsequent merozoite invasion of RBC^DDAO^ and RBC^Violet^. RBC^DDAO^ is readily distinguished from RBC^Violet^ by microscopy (**Figure 1E**) and flow cytometry (**Figure 1F**). SYBR Green I staining allows for the identification of parasitized RBC^DDAO^ (pRBC^DDAO^) and pRBC^Violet^ by both microscopy (**Figure 1D & 1E**) and flow cytometry (**Figure 1G & 1H**). No spectral interference of the three fluorescent dyes was detected by flow cytometry or microscopy. Additionally, we confirmed that dye is not transferred between RBCs during the course of barcoded RBC invasion experiments (**data not shown**). The relative risk of two different target RBC populations to *P. falciparum* invasion was determined with an unadjusted Odds Ratio. We have termed this measurement the Susceptibility Index (**SI**). An SI of 1.0 indicates no difference in the risk of invasion of two target RBC populations. The SI of RBC^DDAO^ and RBC^Violet^ for 3D7, Dd2, and FCR3-FMG parasite invasion was 0.97 (confidence interval [CI] 0.96–0.99), 0.90 (CI 0.89–0.91), and 1.08 (CI 1.06–1.09) respectively (**Figure 2**).

### The barcoded RBC invasion assay allows for the direct comparison of *P. falciparum* invasion into two different RBC populations

**Figure 3:**
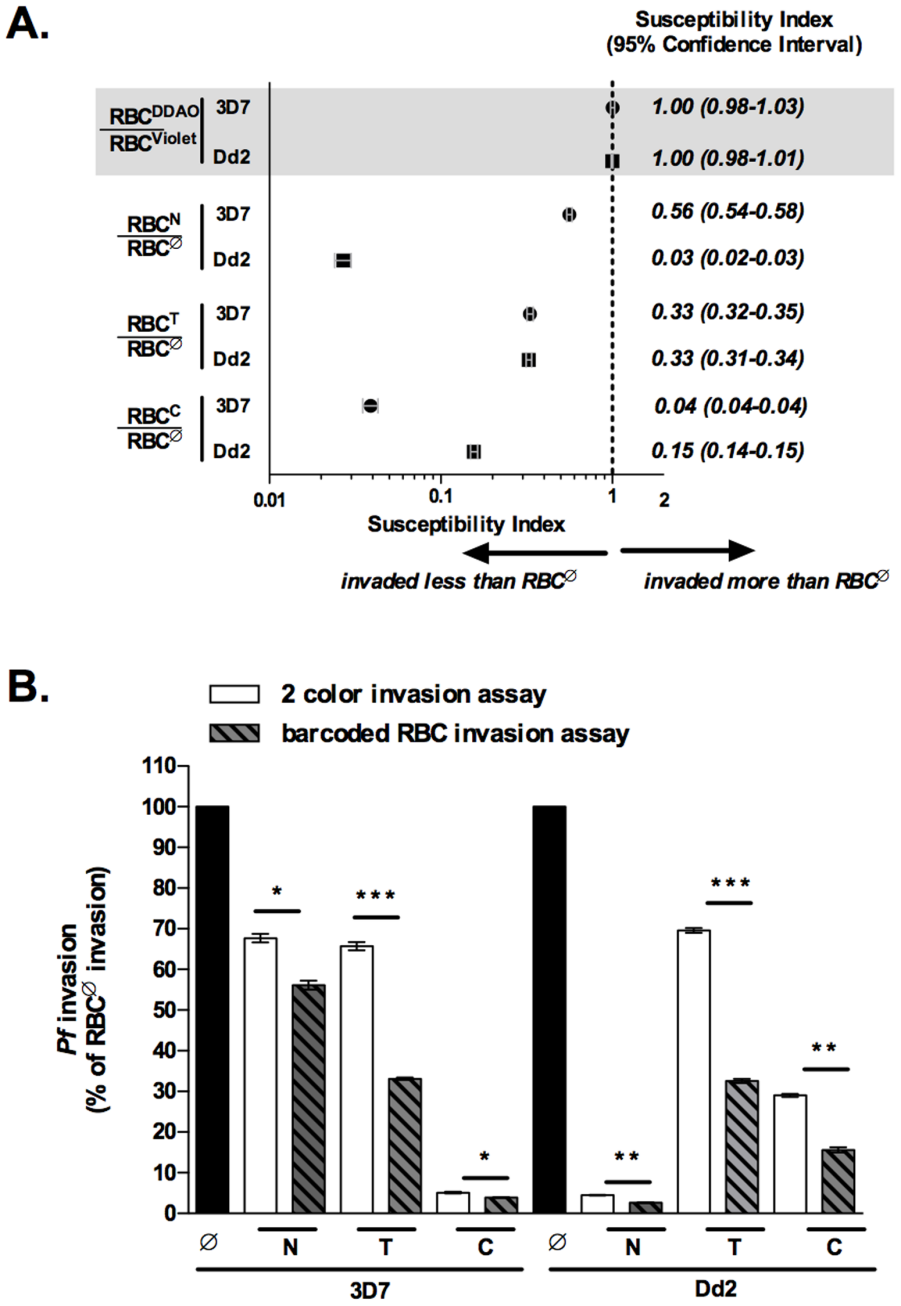
Direct comparison of *P. falciparum* invasion into untreated and enzyme treated RBCs. RBCs were labeled with either CellTrace DDAO or Violet before enzyme treatment (neuraminidase, trypsin, or chymotrypsin). For barcoded RBC invasion assays, 1×10^7^ RBCs of differentially labeled and enzyme treated RBC populations were combined for a total of 2×10^7^ RBCs per well. For two-color invasion assays 2×10^7^ of each RBC population – untreated (RBC^Ø^), neuraminidase (RBC^N^), trypsin (RBC^T^), and chymotrypsin (RBC^C^) – were inoculated into separate wells. Two-color and barcoded RBC invasion assays were inoculated with 2×10^5^ of MACS enriched trophozoite stage 3D7 or Dd2 *P. falciparum* parasites and invasion assays were performed as previously described. (A) SI for 3D7 and Dd2 invasion into barcoded RBC invasion assays containing RBC^Ø^ and either RBC^N^, RBC^T^, or RBC^C^. The marker represents the SI point estimate and the bar represents the 95% CI. (B) 3D7 and Dd2 invasion of RBC^N^, RBC^T^ and RBC^C^ normalized to invasion of RBC^Ø^ for both two-color and barcoded RBC invasion assays. Bars represent the mean and error bars represent the SD. Student's *t*-tests were used to calculate differences in invasion between two-color and barcoded RBC invasion assays, *p<0.005, **p<0.0002, and ***p<7E-6. Data is from a representative experiment of three independent experiments, each performed in triplicate.

Neuraminidase (N), trypsin (T), and chymotrypsin (C) are commonly used to study *P. falciparum* merozoite RBC invasion. Treatment of RBCs with any of these enzymes reduces merozoite RBC invasion. Different *P. falciparum* laboratory strains as well as clinical isolates exhibit different enzyme sensitivities[2]. To demonstrate the functionality of the barcoded RBC invasion assay, we have utilized RBCs treated with these enzymes in combination with *P. falciparum* strains 3D7 and Dd2, which have well characterized invasion phenotypes. Specifically, 3D7 invasion is less sensitive to neuraminidase than Dd2, 3D7 and Dd2 invasion is similarly sensitive to trypsin, and Dd2 invasion is less sensitive to chymotrypsin than 3D7 [17]. Equal numbers of untreated (RBC^Ø^) and either neuraminidase (RBC^N^), trypsin (RBC^T^), or chymotrypsin (RBC^C^) treated RBCs were combined in the barcoded RBC invasion assay and the SI of RBC^Ø^ and enzyme treated RBCs to 3D7 and Dd2 parasite invasion was determined. The SI of RBC^Ø^ and RBC^N^, RBC^T^, and RBC^C^ to 3D7 parasite invasion was 0.56 (CI 0.54–0.58), 0.33 (CI 0.32–0.35), and 0.04 (CI 0.04–0.04) respectively. The SI of RBC^Ø^ and RBC^N^, RBC^T^, and RBC^C^ to Dd2 invasion was 0.03 (CI 0.02–0.03), 0.33 (CI 0.31–0.34), and 0.15 (CI 0.14–0.15) respectively. The SI of all RBC^Ø^ and enzyme treated RBC combinations were significantly different from the control conditions (combination of RBC^DDAO^ and RBC^Violet^) (**Figure 3A**). These results are consistent with previously reported enzyme sensitivities of strains 3D7 and Dd2.

We next compared the two-color [17] and barcoded RBC invasion assays. The two-color invasion assay measures *P. falciparum* invasion of different RBC populations in adjacent but separate culture wells, while the barcoded RBC invasion assay directly compares *P. falciparum* invasion of two different RBC populations in the same culture well. In both assays, the invasion of each parasite strain into RBC^N^, RBC^T^ and RBC^C^ was normalized to invasion into RBC^Ø^. 3D7 and Dd2 parasite sensitivity to all three enzymes was significantly greater in the barcoded RBC than the two-color invasion assay. Specifically, 3D7 and Dd2 invasion into RBC^N^ was 67.7% (SD±1.8) and 4.5% (SD±0.2) in the two-color assay and 56.1% (SD±1.9) and 2.6% (SD±0.7) in the barcoded RBC assay. 3D7 and Dd2 invasion into RBC^T^ was 65.7% (SD±1.8) and 69.6% (SD±1.0) in the two-color assay and 33.1% (SD±0.45) and 32.5% (SD±0.9) in the barcoded RBC assay. Finally, 3D7 and Dd2 invasion into RBC^C^ was 5.1% (SD±0.4) and 32.9% (SD±0.9) in the two-color assay and 3.9% (SD±0.1) and 15.6% (SD±1.1) in the barcoded RBC assay (**Figure 3B**). Together, these results show that the two-color and barcoded RBC assays reveal the same trends but that the barcoded RBC assay is better able to detect small differences in *P. falciparum* invasion.

### The barcoded RBC invasion assay can be used to study the effect of RBC population dynamics on *P. falciparum* infection

**Figure 4:**
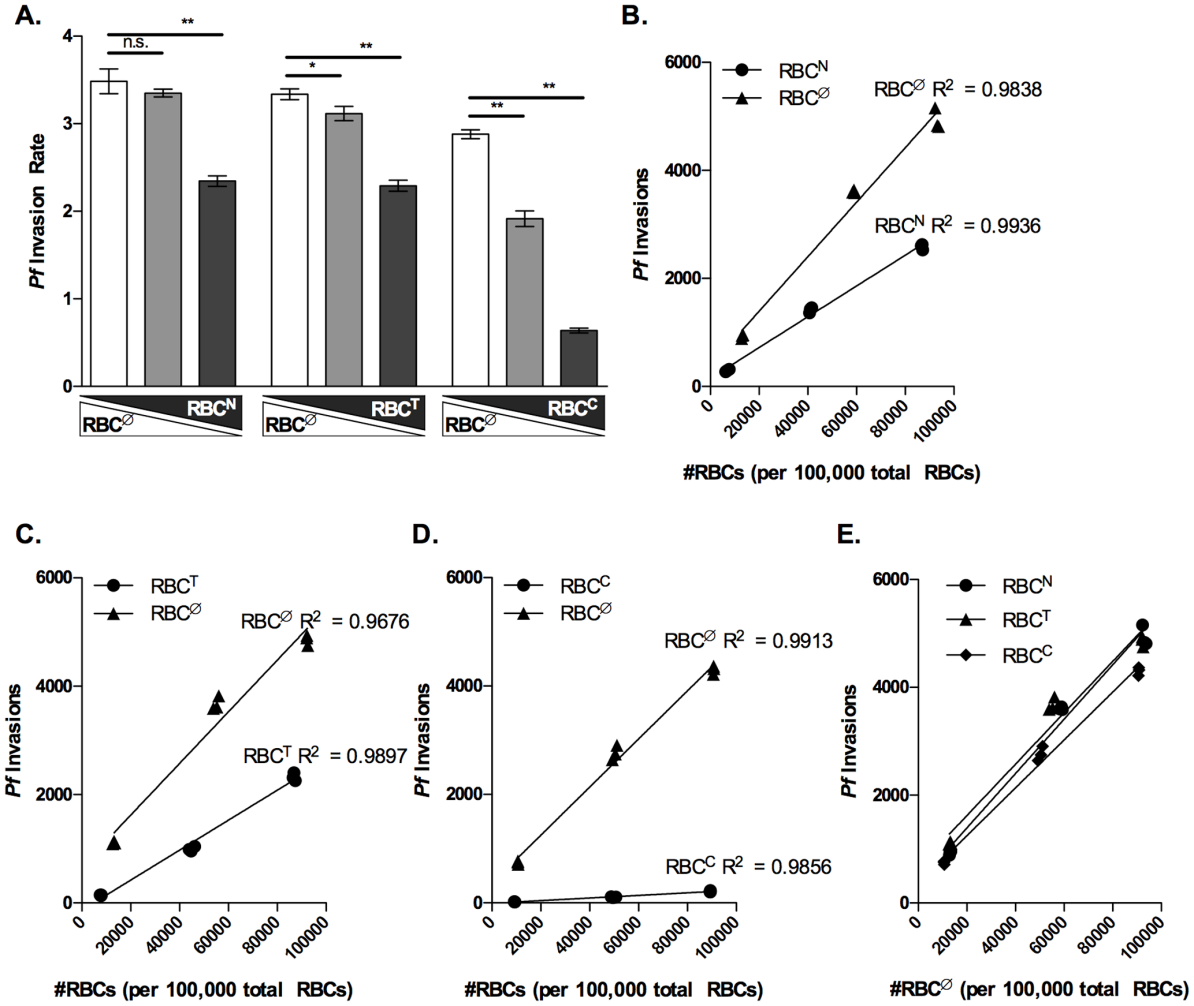
Invasion of *P. falciparum* strain 3D7 decreases as untreated RBCs are replaced with enzyme treated RBCs. RBCs were labeled with either CellTrace DDAO or Violet before enzyme treatment (neuraminidase, trypsin, or chymotrypsin). 1.8×10^7^, 1×10^7^, and 2×10^6^ non-enzymatically treated RBCs (RBC^Ø^) were combined with 2×10^6^, 1×10^7^, and 1.8×10^7^ of enzyme treated RBCs (RBC^N^, RBC^T^, or RBC^C^) respectively to achieve 10∶1, 1∶1, and 1∶10 combinations of RBC^Ø^ to either RBC^N^, RBC^T^, or RBC^C^ in the barcoded RBC invasion assay. Invasion assays were inoculated with 2×10^5^ of MACS purified trophozoite stage *P. falciparum* strain 3D7 parasites and invasion assays were performed as previously described. Data is from a single representative experiment of three independent experiments performed in triplicate. (A) Rate of 3D7 invasion into 1∶10, 1∶1, and 10∶1 combinations of RBC^Ø^ and either RBC^N^, RBC^T^, or RBC^C^. Bars represent the mean invasion rate and error bars represent the SD. Elongated triangles below the X-axis represent the replacement of RBC^Ø^ (white triangle) with either RBC^N^, RBC^T^, or RBC^C^ (gray triangle) in the total RBC population. Student's *t*-tests were used to compare invasion rates, *p<0.02 and **p<0.0002. (B, C, and D) Data shows the number of invasion events into RBC^Ø^ (triangles) and either RBC^N^, RBC^T^, or RBC^C^ (circles) as the frequency of each RBC type increases from 10% to 90% of the total RBC population. Linear regression was used to determine the best fit line for *P. falciparum* invasion of RBC^Ø^, RBC^N^, RBC^T^, and RBC^C^. ANCOVA was performed to compare the slopes of the lines fit to *P. falciparum* invasion of RBC^Ø^, RBC^N^, RBC^T^, and RBC^C^. The null hypothesis was no difference between RBC^Ø^ and either RBC^N^, RBC^T^, or RBC^C^ (H_0_: β_Ø_  =  β_enzyme_, α = 0.05). ANCOVA performed with GraphPad, Prism, v. 5.04, La Jolla, CA calculated a p<0.0001. (E) RBC^Ø^ datum from panels B–D were superimposed to compare invasion into RBC^Ø^ when RBC^Ø^ was combined with RBC^N^ (circles) RBC^T^ (triangles) or RBC^C^ (diamonds). Linear regression was used to determine the best fit line for RBC^Ø^ invasion data. ANCOVA was performed to compare the slopes of the lines fit to *P. falciparum* invasion of RBC^Ø^. The null hypothesis was that there would be no difference in the invasion of RBC^Ø^ in the presence of the other RBC populations (H_0_: β_Ø_  =  β_enzyme_, α = 0.05). ANCOVA performed with GraphPad, Prism, v. 5.04, La Jolla, CA calculated a p<0.2599.

To study the effect of RBC population dynamics on *P. falciparum* invasion, we replaced 10%, 50%, and 90% of RBC^Ø^ with RBC^N^, RBC^T^, or RBC^C^ and assessed *P. falciparum* invasion. The SI reflects the relative invasion of *P. falciparum* into two different RBC populations. However, the SI does not reflect the total invasion capacity of *P. falciparum*. Therefore for RBC^Ø^ replacement experiments, we examined the invasion rate of *P. falciparum* strains 3D7 and Dd2 into wells containing different combination of RBC^Ø^ and enzyme treated RBC. The invasion rate of both 3D7 and Dd2 parasites decreased significantly as RBC^Ø^ decreased from 90% to 10% and the enzyme treated RBC population (RBC^N^, RBC^T^, or RBC^C^) increased from 10% to 90% of the total RBC population (**Figure 4A** **and** **5A**). Parasite invasion did not significantly change for control conditions in which RBC^Violet^ were replaced with RBC^DDAO^ (**Figure S2**).

**Figure 5:**
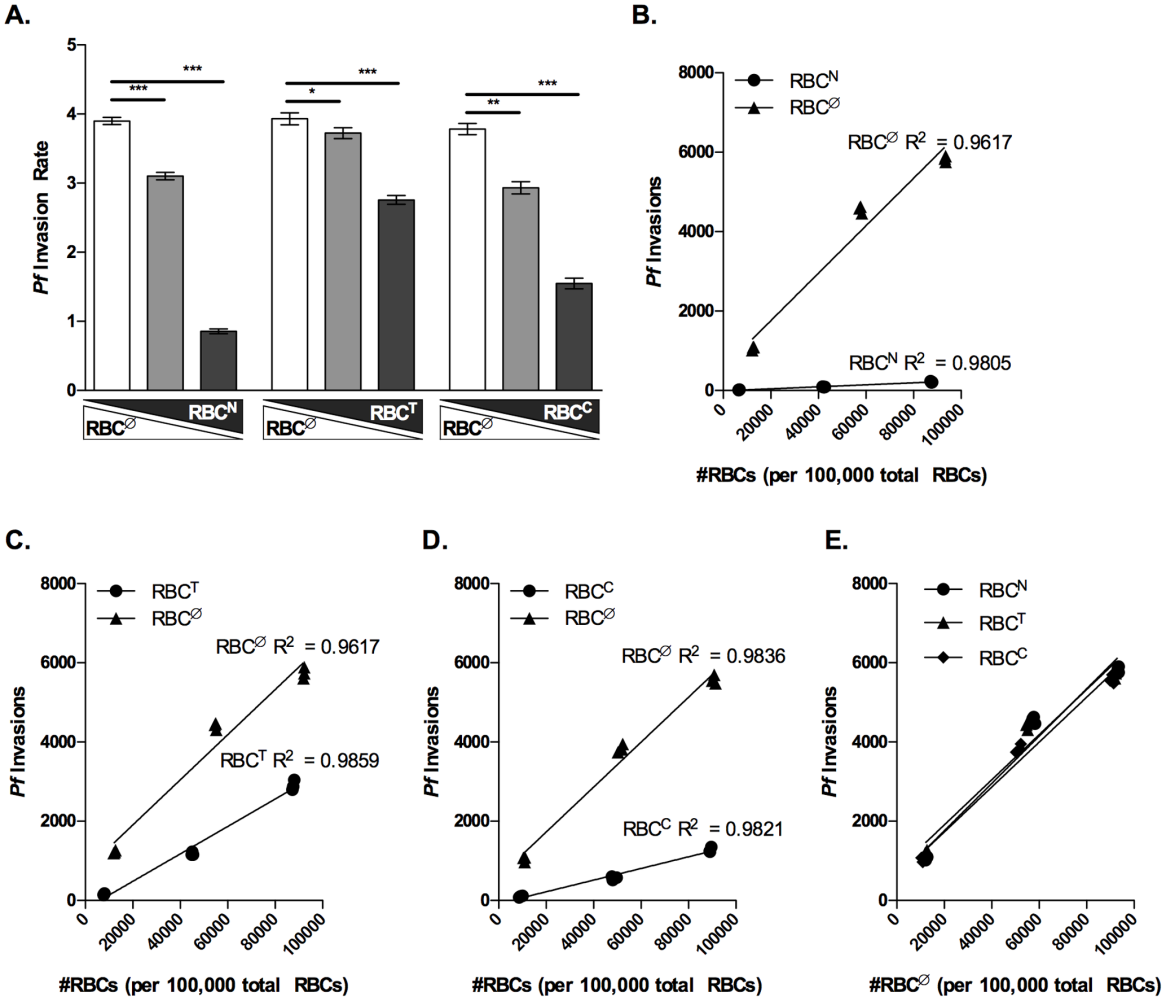
Invasion of *P. falciparum* strain Dd2 decreases as untreated RBCs are replaced with enzyme treated RBCs. RBCs were labeled with either CellTrace DDAO or Violet before enzyme treatment (neuraminidase, trypsin, or chymotrypsin). 1.8×10^7^, 1×10^7^, and 2×10^6^ non-enzymatically treated RBCs (RBC^Ø^) were combined with 2×10^6^, 1×10^7^, and 1.8×10^7^ of enzyme treated RBCs (RBC^N^, RBC^T^, or RBC^C^) respectively to achieve 10∶1, 1∶1, and 1∶10 combinations of RBC^Ø^ to either RBC^N^, RBC^T^, or RBC^C^ in the barcoded RBC invasion assay. Invasion assays were inoculated with 2×10^5^ of MACS purified trophozoite stage *P. falciparum* strain Dd2 parasites and invasion assays were performed as previously described. Data is from a single representative experiment of three independent experiments performed in triplicate. (A) Rate of Dd2 invasion into 1∶10, 1∶1, and 10∶1 combinations of RBC^Ø^ and either RBC^N^, RBC^T^, or RBC^C^. Bars represent the mean invasion rate and error bars represent the SD. Elongated triangles below the X-axis represent the replacement of RBC^Ø^ (white triangle) with either RBC^N^, RBC^T^, or RBC^C^ (gray triangle) in the total RBC population. Student's *t*-tests were used to compare invasion rates, *p<0.04, **p<0.0003, and ***p<5E-5. (B, C, and D) Data shows the number of invasion events into RBC^Ø^ (triangles) and either RBC^N^, RBC^T^, or RBC^C^ (circles) as the frequency of each RBC type increases from 10% to 90% of the total RBC population. Linear regression was used to determine the best fit line for *P. falciparum* invasion of RBC^Ø^, RBC^N^, RBC^T^, and RBC^C^. ANCOVA was performed to compare the slopes of the lines fit to *P. falciparum* invasion of RBC^Ø^, RBC^N^, RBC^T^, and RBC^C^. The null hypothesis was no difference between RBC^Ø^ and either RBC^N^, RBC^T^, or RBC^C^ (H_0_: β_Ø_  =  β_enzyme_, α = 0.05). ANCOVA performed with GraphPad, Prism, v. 5.04, La Jolla, CA calculated a p<0.0002. (E) RBC^Ø^ data from panels B–D were superimposed to compare invasion into RBC^Ø^ when RBC^Ø^ was combined with RBC^N^ (circles) RBC^T^ (triangles) or RBC^C^ (diamonds). Linear regression was used to determine the best fit line for RBC^Ø^ invasion data. ANCOVA was performed to compare the slopes of the lines fit to *P. falciparum* invasion of RBC^Ø^. The null hypothesis was that there would be no difference in the invasion of RBC^Ø^ in the presence of the other RBC populations (H_0_: β_Ø_  =  β_enzyme_, α = 0.05). ANCOVA performed with GraphPad, Prism, v. 5.04, La Jolla, CA calculated a p<0.8335.

Utilizing the ability to distinguish RBC^Ø^ and enzyme treated RBCs provided by the barcoded RBC invasion assay, we next characterized the invasion of *P. falciparum* strains 3D7 and DD2 into RBC^Ø^ and RBC^N^, RBC^T^, and RBC^C^ as the number of each increased in the total RBC population. We observed that the number of 3D7 invasions into all RBC populations increased linearly as each increased in frequency from 10% to 90% of the total RBC population, and that 3D7 invasion as a function of either RBC^N^, RBC^T^, or RBC^C^ number was significantly less than that of RBC^Ø^, p<0.0001 (**Figure 4B, 4C, and 4D**). Similar analysis of Dd2 invasion of RBC^Ø^, RBC^N^ RBC^T^, and RBC^C^ also increased linearly as each increased from 10% to 90% of the total RBC population. As with 3D7 invasion, Dd2 invasion as a function of either RBC^N^, RBC^T^, or RBC^C^ number was significantly less than that of RBC^Ø^, p<0.0002 (**Figure 5B, 5C, and 5D**). Together this data demonstrates the capacity of the barcoded RBC invasion assay to study changing RBC population dynamics and *P. falciparum* invasion.

Finally, we investigated the hypothesis that the presence of one RBC population may impact the susceptibility of another to *P. falciparum* invasion. To determine whether the presence of RBC^N^, RBC^T^, or RBC^C^ affects 3D7 or Dd2 invasion into RBC^Ø^, we compared 3D7 and Dd2 invasion of RBC^Ø^ when RBC^Ø^ were replaced with either RBC^N^, RBC^T^, or RBC^C^ and the total number of RBCs remained constant. We observed that the number of 3D7 and Dd2 invasions into RBC^Ø^ was not affected by the presence of RBC^N^, RBC^T^, or RBC^C^ (**Figure 4E** **and** **5E**).

## Discussion

In the present study we have coupled RBC barcoding and flow cytometry to develop a *P. falciparum* invasion assay in which parasite invasion of two RBC populations is directly compared. The use of CellTrace dyes to label or “barcode” cell populations was developed to perform high-throughput drug screening and signal profiling [20]. Here, we demonstrate the first application of this approach to “barcode” RBC populations with the aim of studying *P. falciparum* invasion within a heterogeneous population.

We've demonstrated that RBCs labeled with CellTrace dyes DDAO and Violet and subsequently combined can be definitively identified by both microscopy and flow cytometry. Furthermore, we demonstrate that parasitized DDAO and Violet RBCs can be identified with SYBR Green I staining (**Figure 1**). With the introduction of the susceptibly index (SI) as a robust tool for analyzing the relative risk of two RBC populations to *P. falciparum* invasion, we proceeded to demonstrate that DDAO and Violet RBCs are similarly susceptible to *P. falciparum* strains 3D7, Dd2, and FCR3-FMG invasion (**Figure 2**).

Having established that *P. falciparum* invasion of two RBC populations may be compared directly by barcoding RBCs with CellTrace dyes, we proceeded to pursue the power of the barcoded RBC invasion assay by directly comparing *P. falciparum* invasion into untreated and enzyme treated RBCs. The effect of enzymatically treating RBCs with neuraminidase, trypsin, and chymotrypsin on *P. falciparum* invasion has been well studied [21] and therefore served as an ideal system for validating the barcoded RBC invasion assay. Direct comparison of *P. falciparum* (strains 3D7 and Dd2) invasion of RBC^Ø^ and either RBC^N^, RBC^T^, or RBC^C^ with the barcoded RBC invasion assay resulted in the same invasion trends as previously described [17]. Moreover, we observed 3D7 and Dd2 sensitivity to all three enzymes to be significantly greater in the barcoded RBC invasion assay (direct measure of invasion) as compared to the two-color invasion assay (indirect measure of invasion) (**Figure 3**). This is consistent with Pattanapanyasat et al. 's conclusion that direct comparison of parasite growth allows for greater sensitivity in detecting small differences in growth rates between physiologically different RBCs than independent growth rate assessment [13]. Assay sensitivity is very important to consider as there is diversity in both host RBC and parasite factors which interact to shape malaria pathogenesis [4], [22]. In the case of merozoite RBC invasion, the redundancy in parasite invasion pathways increases the necessity of sensitive methods [23]–[27].

The RBC is essential for the erythrocytic stage of malaria infection; consequentially human RBC traits, such as hemoglobinopathies [22], G6PD deficiency [28] and differential expression of host RBC invasion ligands [29], have arisen in the human population providing protection from malaria. In addition, RBC age as well as other potential factors such as iron deficiency, oxidative stress, and heterozygous G6PD deficiency result in heterogeneity in a single individual's RBC population [30]. As RBC age, oxidative damage, and G6PD enzyme levels are all known to impact erythrocyte stage *P. falciparum* infection [15]–[18], it is likely that the changing dynamics of an individual's RBC population has the potential to dramatically impact the course of the disease [31]. The barcoded RBC invasion assay introduces the first method for studying the effect of changing RBC populations on *P. falciparum* infection. In the present study, we demonstrate that replacing a susceptible RBC population (RBC^Ø^) with a less susceptible RBC population (enzyme treated RBCs) reduces the invasion rate of *P. falciparum* (**Figure 4A** **and** **5A**). Furthermore, barcoding allowed for the quantitation of merozoite invasion events into RBC^Ø^ and enzyme treated RBC populations as the number of each increased in the total RBC population. We show the number of *P. falciparum* invasion events into RBC^Ø^ and enzyme treated RBCs is linearly related to the RBC number. As would be expected, *P. falciparum* invasion of RBC^Ø^ and enzyme treated RBCs as a function of RBC number is significantly different (**Figure 4B–D** **and** **5B–D**). Finally, we show that *P. falciparum* invasion of untreated RBCs is not competitive as it is not affected by the presence of enzyme treated RBCs (**Figure 4E** **and** **5E**).

In conclusion, we have developed a sensitive, high-throughput experimental assay for directly comparing the susceptibility of different RBC populations to *P. falciparum* merozoite invasion. We additionally demonstrate how the barcoded RBC invasion assay may be utilized to study the impact of changing RBC population dynamics on overall *P. falciparum* invasion. Furthermore, by using different Celltrace dyes and/or different concentrations of a given dye, the RBC barcoding approach is easily modifiable and could be expanded to study multiple RBC populations simultaneously. Future experimentation using this approach will provide invaluable insight into the relationship between *P. falciparum* and its human host.

## Supporting Information

Figure S1 **Barcoding RBCs with CellTrace Violet does not impact** ***P. falciparum*** **invasion.** An equal number of RBC^Violet^ and RBC^unlabeled^ (1×10^7^) were combined for a total of 2×10^7^ uninfected RBCs per well, and inoculated with 2×10^5^ MACS purified trophozoite stage *P. falciparum* strains 3D7, Dd2 or FCR3-FMG. Invasion experiments were incubated 18–24 hours to allow for rupture of schizonts and subsequent invasion of merozoites into labeled RBCs, then stained with DNA dye SYBR Green I (to identify pRBCs), fixed and analyzed by flow cytometry. The SI, an unadjusted odds ratio assessing the relative risk of RBC^Violet^ and RBC^unlabeled^ to 3D7 and Dd2 invasion, was determined. The marker represents the SI point estimate and the bar represents the 95% confidence interval (CI). Data is from a representative experiment of three independent experiments, each performed in triplicate.
(TIF)

Figure S2 **Effect of replacing CellTrace Violet barcoded RBCs with DDAO barcoded RBCs on** ***P. falciparum*** **strain 3D7 and Dd2 invasion.** RBCs were labeled with either CellTrace DDAO or Violet and 1.8×10^7^, 1×10^7^, and 2×10^6^ Violet labeled RBCs (RBC^Violet^) were combined with 2×10^6^, 1×10^7^, and 1.8×10^7^ of DDAO labeled RBCs (RBC^DDAO^) to achieve 10∶1, 1∶1, and 1∶10 combinations of RBC^Violet^ to RBC^DDAO^ in the barcoded RBC invasion assay. Invasion assays were inoculated with 2×10^5^ of MACS purified trophozoite stage *P. falciparum* strain 3D7 or Dd2 parasites and invasion assays were performed as previously described. Data is from a single representative experiment of three independent experiments performed in triplicate. (A) Rate of 3D7 and Dd2 invasion into 1∶10, 1∶1, and 10∶1 combinations of RBC^Violet^ and RBC^DDAO^. Bars represent the mean invasion rate and error bars represent the SD. Elongated triangles below the X-axis represent the replacement of RBC^Violet^ (white triangle) with RBC^DDAO^ (gray triangle) in the total RBC population. (B and C) Data shows the number of 3D7 (B) and Dd2 (C) invasion events into RBC^Violet^ (triangles) and RBC^DDAO^ (circles) as the frequency of each RBC type increases from 10% to 90% of the total RBC population. Linear regression was used to determine the best fit line for *P. falciparum* invasion of RBC^Violet^ and RBC^DDAO^. ANCOVA was performed to compare the slopes of the lines fit to *P. falciparum* invasion of RBC^Violet^ and RBC^DDAO^. The null hypothesis was no difference between RBC^Ø^ and either RBC^N^, RBC^T^, or RBC^C^ (H_0_: β_Ø_  =  β_enzyme_, α = 0.05). ANCOVA performed with GraphPad, Prism, v. 5.04, La Jolla, CA calculated a p<0.0001. and p<0.06 for 3D7 and Dd2 respectively.
(TIF)

## References

[1] WHO | World Malaria Report 2011 (n.d.). WHO. Available: http://www.who.int/malaria/world_malaria_report_2011/en/. Accessed 2012 August 13.

[2] Bei AK, Duraisingh MT (2012) Functional analysis of erythrocyte determinants of Plasmodium infection. Int J Parasitol 42: 575–582. 22726752 10.1016/j.ijpara.2012.03.006 PMC3383627

[3] Boyle MJ, Wilson DW, Beeson JG (2013) New approaches to studying Plasmodium falciparum merozoite invasion and insights into invasion biology. Int J Parasitol 43: 1–10. 23220090 10.1016/j.ijpara.2012.11.002

[4] Wright GJ, Rayner JC (2014) Plasmodium falciparum erythrocyte invasion: combining function with immune evasion. PLoS Pathog Mar 20 10(3): e1003943. 10.1371/journal.ppat.1003943 PMC3961354 24651270

[5] Brandão MM, Castro M de LRB, Fontes A, Cesar CL, Costa FF, et al (2009) Impaired red cell deformability in iron deficient subjects. Clin Hemorheol Microcirc 43: 217–221. 19847056 10.3233/CH-2009-1211

[6] Nagababu E, Gulyani S, Earley CJ, Cutler RG, Mattson MP, et al (2008) Iron-Deficiency Anemia Enhances Red Blood Cell Oxidative Stress. Free Radic Res 42: 824–829. 19051108 10.1080/10715760802459879 PMC2730642

[7] Gifford SC, Derganc J, Shevkoplyas SS, Yoshida T, Bitensky MW (2006) A detailed study of time-dependent changes in human red blood cells: from reticulocyte maturation to erythrocyte senescence. Br J Haematol 135: 395–404. 16989660 10.1111/j.1365-2141.2006.06279.x

[8] Franco RS, Puchulu-Campanella ME, Barber LA, Palascak MB, Joiner CH, et al (2013) Changes in the properties of normal human red blood cells during in vivo aging. Am J Hematol 88: 44–51. 23115087 10.1002/ajh.23344 PMC4067949

[9] Cordero JF, Rodríguez PJ, Romero PJ (2004) Differences in intramembrane particle distribution in young and old human erythrocytes. Cell Biol Int 28: 423–431. 15223018 10.1016/j.cellbi.2004.03.002

[10] Rowe JA, Opi DH, Williams TN (2009) Blood groups and malaria: fresh insights into pathogenesis and identification of targets for intervention. Curr Opin Hematol 16: 480–487. 19812491 10.1097/MOH.0b013e3283313de0 PMC2878475

[11] Walker PS, Reid ME (2010) The Gerbich blood group system: a review. Immunohematol Am Red Cross 26: 60–65. 20932076

[12] Shapiro HM, Apte SH, Chojnowski GM, Hänscheid T, Rebelo M, et al (2013) Cytometry in malaria-a practical replacement for microscopy? Curr Protoc Cytom Editor Board J Paul Robinson Manag Ed Al Chapter 11: Unit11.20. 10.1002/0471142956.cy1120s65 23835802

[13] Pattanapanyasat K, Yongvanitchit K, Heppner DG, Tongtawe P, Kyle DE, et al (1996) Culture of malaria parasites in two different red blood cell populations using biotin and flow cytometry. Cytometry 25: 287–294. 8914826 10.1002/(SICI)1097-0320(19961101)25:3<287::AID-CYTO10>3.0.CO;2-S

[14] Pattanapanyasat K, Yongvanitchit K, Tongtawe P, Tachavanich K, Wanachiwanawin W, et al (1999) Impairment of Plasmodium falciparum Growth in Thalassemic Red Blood Cells: Further Evidence by Using Biotin Labeling and Flow Cytometry. Blood 93: 3116–3119. 10216109

[15] Breuer WV, Ginsburg H, Cabantchik ZI (1983) An assay of malaria parasite invasion into human erythrocytes. The effects of chemical and enzymatic modification of erythrocyte membrane components. Biochim Biophys Acta 755: 263–271. 6338931 10.1016/0304-4165(83)90213-1

[16] Bei AK, Desimone TM, Badiane AS, Ahouidi AD, Dieye T, et al (2010) A flow cytometry-based assay for measuring invasion of red blood cells by Plasmodium falciparum. Am J Hematol 85: 234–237. 20196166 10.1002/ajh.21642 PMC3089760

[17] Theron M, Hesketh RL, Subramanian S, Rayner JC (2010) An adaptable two-color flow cytometric assay to quantitate the invasion of erythrocytes by Plasmodium falciparum parasites. Cytom Part J Int Soc Anal Cytol 77: 1067–1074. 10.1002/cyto.a.20972 PMC3047707 20872885

[18] Ribaut C, Berry A, Chevalley S, Reybier K, Morlais I, et al (2008) Concentration and purification by magnetic separation of the erythrocytic stages of all human Plasmodium species. Malar J 7: 45. 18321384 10.1186/1475-2875-7-45 PMC2292734

[19] Clark M, Fisher NC, Kasthuri R, Cerami Hand C (2013) Parasite maturation and host serum iron influence the labile iron pool of erythrocyte stage Plasmodium falciparum. Br J Haematol 161(2): 262–9. 23398516 10.1111/bjh.12234 PMC4249674

[20] Krutzik PO, Nolan GP (2006) Fluorescent cell barcoding in flow cytometry allows high-throughput drug screening and signaling profiling. Nat Methods 3: 361–368. 16628206 10.1038/nmeth872

[21] Cowman AF, Berry D, Baum J (2012) The cellular and molecular basis for malaria parasite invasion of the human red blood cell. J Cell Biol 198: 961–971. 22986493 10.1083/jcb.201206112 PMC3444787

[22] Taylor SM, Cerami C, Fairhurst RM (2013) Hemoglobinopathies: slicing the Gordian knot of Plasmodium falciparum malaria pathogenesis. PLoS Pathog 9: : e1003327. 9(5): : e1003327. Epub 2013 May 16. 10.1371/journal.ppat.1003327 PMC3656091 23696730

[23] Badiane AS, Bei AK, Ahouidi AD, Patel SD, Salinas N, et al (2013) Inhibitory humoral responses to the Plasmodium falciparum vaccine candidate EBA-175 are independent of the erythrocyte invasion pathway. Clin Vaccine Immunol CVI 20: 1238–1245. 23761656 10.1128/CVI.00135-13 PMC3754519

[24] Lantos PM, Ahouidi AD, Bei AK, Jennings CV, Sarr O, et al (2009) Erythrocyte invasion profiles are associated with a common invasion ligand polymorphism in Senegalese isolates of Plasmodium falciparum. Parasitology 136: 1–9. 19126266 10.1017/S0031182008005167 PMC12743632

[25] Baum J, Pinder M, Conway DJ (2003) Erythrocyte invasion phenotypes of Plasmodium falciparum in The Gambia. Infect Immun 71: 1856–1863. 12654801 10.1128/IAI.71.4.1856-1863.2003 PMC152018

[26] Lobo C-A, Rodriguez M, Struchiner CJ, Zalis MG, Lustigman S (2006) Associations between defined polymorphic variants in the PfRH ligand family and the invasion pathways used by P. falciparum field isolates from Brazil. Mol Biochem Parasitol 149: 246–251. 16857278 10.1016/j.molbiopara.2006.05.011

[27] Lopez-Perez M, Villasis E, Machado RLD, Póvoa MM, Vinetz JM, et al. (2012) Plasmodium falciparum Field Isolates from South America Use an Atypical Red Blood Cell Invasion Pathway Associated with Invasion Ligand Polymorphisms. PloS One 7(10): e47913. Epub 2012 Oct 31. 10.1371/journal.pone.0047913 PMC3485327 23118907

[28] Roth EF Jr, Raventos-Suarez C, Rinaldi A, Nagel RL (1983) Glucose-6-phosphate dehydrogenase deficiency inhibits in vitro growth of Plasmodium falciparum. Proc Natl Acad Sci U S A 80: 298–299. 6337374 10.1073/pnas.80.1.298 PMC393360

[29] Tham W-H, Wilson DW, Lopaticki S, Schmidt CQ, Tetteh-Quarcoo PB, et al (2010) Complement receptor 1 is the host erythrocyte receptor for Plasmodium falciparum PfRh4 invasion ligand. Proc Natl Acad Sci U S A 107: 17327–17332. 20855594 10.1073/pnas.1008151107 PMC2951459

[30] Sansone G, Rasore-Quartino A, Veneziano G (1963) Demonstration on blood smears of a double erythrocyte population in women heterogeneous for glucose-6-Phosphate dehydrogenase deficiency. Pathologica 55: 371–375. 14122883

[31] Cromer D, Stark J, Davenport MP (2009) Low red cell production may protect against severe anemia during a malaria infection—insights from modeling. J Theor Biol 257: 533–542. 19168080 10.1016/j.jtbi.2008.12.019

