# Supplementary figures and images for: RBC Barcoding Allows for the Study of Erythrocyte Population Dynamics and P. falciparum Merozoite Invasion
Source: PLoS One. 2014 Jul 1;9(7):e101041. doi: 10.1371/journal.pone.0101041 (PMC4077748; doi:10.1371/journal.pone.0101041)

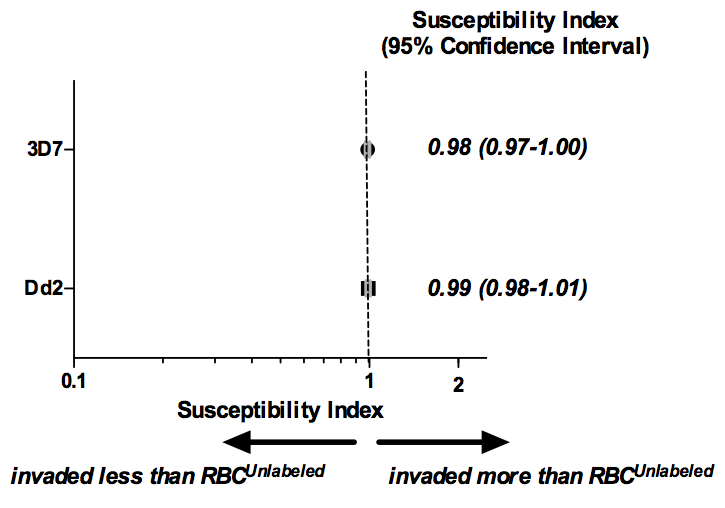

Supplement: Figure S1 — Barcoding RBCs with CellTrace Violet does not impact P. falciparum invasion. An equal number of RBCViolet and RBCunlabeled (1×10^7^) were combined for a total of 2×10^7^ uninfected RBCs per well, and inoculated with 2×10^5^ MACS purified trophozoite stage P. falciparum strains 3D7, Dd2 or FCR3-FMG. Invasion experiments were incubated 18–24 hours to allow for rupture of schizonts and subsequent invasion of merozoites into labeled RBCs, then stained with DNA dye SYBR Green I (to identify pRBCs), fixed and analyzed by flow cytometry. The SI, an unadjusted odds ratio assessing the relative risk of RBCViolet and RBCunlabeled to 3D7 and Dd2 invasion, was determined. The marker represents the SI point estimate and the bar represents the 95% confidence interval (CI). Data is from a representative experiment of three independent experiments, each performed in triplicate. (TIF) [file pone.0101041.s001.tif]

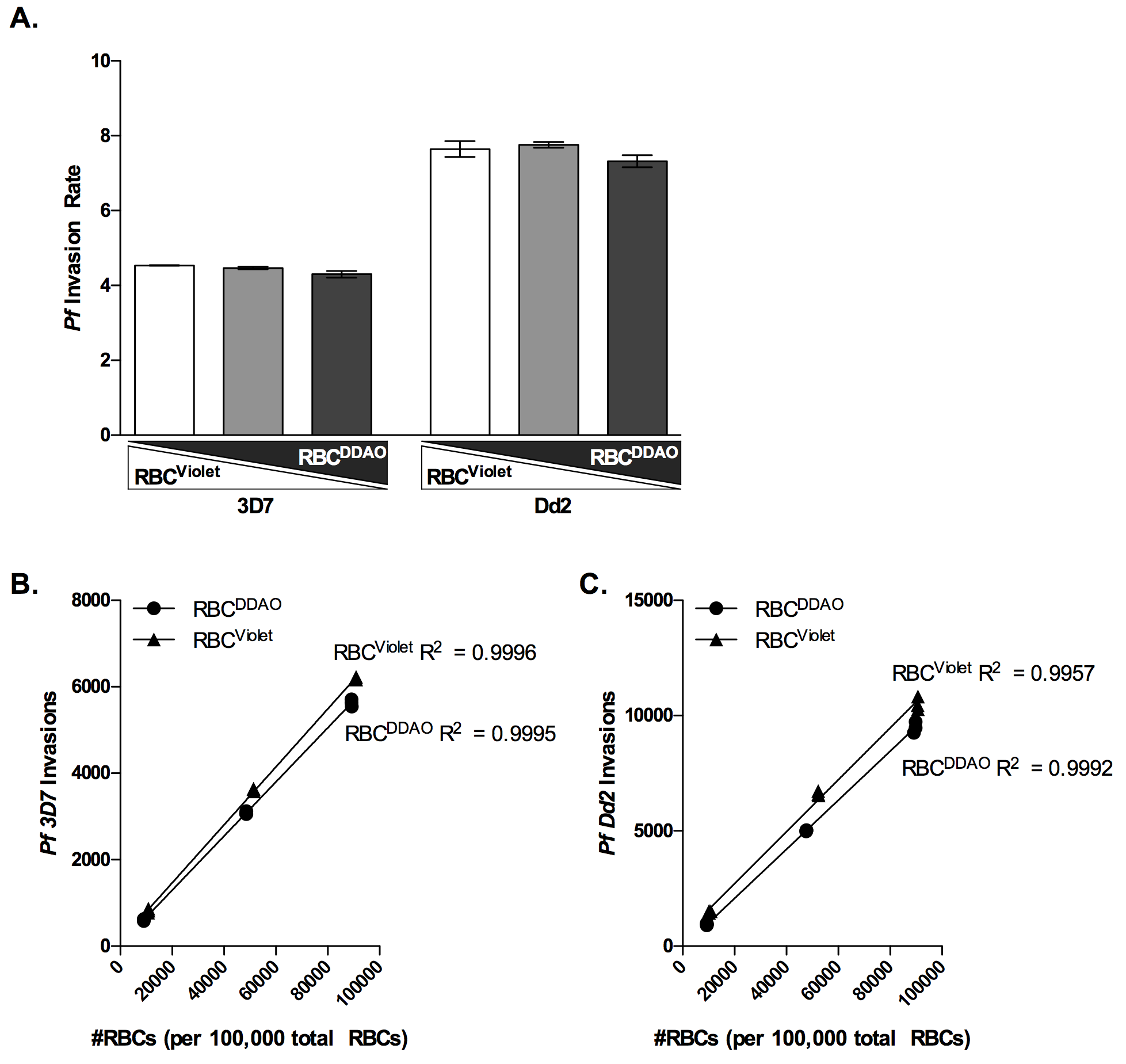

Supplement: Figure S2 — Effect of replacing CellTrace Violet barcoded RBCs with DDAO barcoded RBCs on P. falciparum strain 3D7 and Dd2 invasion. RBCs were labeled with either CellTrace DDAO or Violet and 1.8×10^7^, 1×10^7^, and 2×10^6^ Violet labeled RBCs (RBCViolet) were combined with 2×10^6^, 1×10^7^, and 1.8×10^7^ of DDAO labeled RBCs (RBCDDAO) to achieve 10∶1, 1∶1, and 1∶10 combinations of RBCViolet to RBCDDAO in the barcoded RBC invasion assay. Invasion assays were inoculated with 2×10^5^ of MACS purified trophozoite stage P. falciparum strain 3D7 or Dd2 parasites and invasion assays were performed as previously described. Data is from a single representative experiment of three independent experiments performed in triplicate. (A) Rate of 3D7 and Dd2 invasion into 1∶10, 1∶1, and 10∶1 combinations of RBCViolet and RBCDDAO. Bars represent the mean invasion rate and error bars represent the SD. Elongated triangles below the X-axis represent the replacement of RBCViolet (white triangle) with RBCDDAO (gray triangle) in the total RBC population. (B and C) Data shows the number of 3D7 (B) and Dd2 (C) invasion events into RBCViolet (triangles) and RBCDDAO (circles) as the frequency of each RBC type increases from 10% to 90% of the total RBC population. Linear regression was used to determine the best fit line for P. falciparum invasion of RBCViolet and RBCDDAO. ANCOVA was performed to compare the slopes of the lines fit to P. falciparum invasion of RBCViolet and RBCDDAO. The null hypothesis was no difference between RBCØ and either RBCN, RBCT, or RBCC (H0: βØ = βenzyme, α = 0.05). ANCOVA performed with GraphPad, Prism, v. 5.04, La Jolla, CA calculated a p<0.0001. and p<0.06 for 3D7 and Dd2 respectively. (TIF) [file pone.0101041.s002.tif]
